# Abrogation of mercuric chloride-induced nephritis in the Brown Norway rat by treatment with antibodies against TNFα

**DOI:** 10.1155/S0962935195000718

**Published:** 1995-11

**Authors:** A. Molina, F. Sánchez-Madrid, T. Bricio, A. Martín, E. Escudero, V. Alvarez, F. Mampaso

**Affiliations:** 1 Department of Pathology Hospital Ramón y Cajal Madrid Spain; 2 Immunology Section Hospital de la Princesa Madrid Spain

## Abstract

HgCl_2_ induces an autoimmune disease in the Brown Norway rat characterized by synthesis of autoantibodies (mainly, anti-GBM Abs), severe proteinuria and interstitial nephritis. Also, HgCl_2_- injected rats develop glomerular cell infiltrates consisting of ED1^+^ cells (monocyte/macrophage), starting on day 4 and reaching a maximum on day 8. Treatment with anti-TNF-α antiserum had preventative effects as it reduced the urinary protein levels to close to the normal range and also blocked the influx of inflammatory cells in the renal glomeruli and interstitium, but circulating anti-GBM and lineal glomerular IgG deposits were unmodified. In addition, whole isolated glomeruli from HgCl_2_-induced nephritis secreted TNF-α commencing on day 8, being maximally detected on day 11 and preceding, between 2 to 3 days, the development of proteinuria. The administration of anti-TNF-α antiserum or anti-α4 integrin mAb completely abrogated the synthesis of TNF-α in glomeruli isolated from the respective treated groups of animals, in addition to the proteinuria. Taken together our results confirm that TNF-α plays an important role in the induction and development of HgCl_2_-induced nephritis and highlights the pathogenic importance of the local release of TNF in those renal diseases in which prominent glomerular macrophage accumulation is a constant feature.


Mediators of Inflammation 4, 444–451 (1995)

### Abrogation of mercuric chloride-induced nephritis in the Brown Norway rat by treatment with antibodies against TNFα

A. Molina,[1] F. Sánchez-Madrid,[2] T. Bricio,[1] A. Martín,[1] E. Escudero,[1] V. Alvarez[2] and F. Mampaso[1,CA]

[1]Department of Pathology, Hospital Ramón y Cajal, Madrid, and [2]Immunology Section, Hospital de la Princesa, Madrid, Spain

[CA]Corresponding Author

**$HgCl_2$ induces an autoimmune disease in the Brown Norway rat characterized by synthesis of autoantibodies (mainly, anti-GBM Abs), severe proteinuria and interstitial nephritis. Also, $HgCl_2$-injected rats develop glomerular cell infiltrates consisting of $ED1^+$ cells (monocyte/macrophage), starting on day 4 and reaching a maximum on day 8. Treatment with anti-TNF-α antiserum had preventative effects as it reduced the urinary protein levels to close to the normal range and also blocked the influx of inflammatory cells in the renal glomeruli and interstitium, but circulating anti-GBM and lineal glomerular IgG deposits were unmodified. In addition, whole isolated glomeruli from $HgCl_2$-induced nephritis secreted TNF-α commencing on day 8, being maximally detected on day 11 and preceding, between 2 to 3 days, the development of proteinuria. The administration of anti-TNF-α antiserum or anti-α4 integrin mAb completely abrogated the synthesis of TNF-α in glomeruli isolated from the respective treated groups of animals, in addition to the proteinuria. Taken together our results confirm that TNF-α plays an important role in the induction and development of $HgCl_2$-induced nephritis and highlights the pathogenic importance of the local release of TNF in those renal diseases in which prominent glomerular macrophage accumulation is a constant feature.**

**Key words:** α4-Integrin, glomerular macrophages, immunosuppression, mercury chloride, nephritis, proteinuria, TNF-α

### Introduction

Mercuric chloride ($HgCl_2$) induces an autoimmune disease in the Brown Norway (BN) rat[1] mediated by T-dependent polyclonal B-cell activation[2] resulting in hypergammaglobulinaemia, synthesis of anti-nuclear and anti-glomerular basement membrane (GBM) autoantibodies as well as development of nephritis with glomerular lineal deposits of Ig and proteinuria.[1–3] The histological renal lesions consist of a transient influx of mononuclear cells (mainly MHC class II-bearing T-lymphocytes and monocytes) into the renal interstitium and monocytes and $CD8^+$ T lymphocytes into the glomeruli.[4]

It has been shown recently that interaction between lymphocytes and endothelial cells (EC) is crucial in the development of this renal disease.[5] Treatment with Abs against the α4-integrin abrogated the development of interstitial nephritis and virtually abolished the anti-GBM Abs production. Consequently, glomerular deposition of anti-GBM Abs was absent and proteinuria was reduced to levels close to the normal range. In contrast, anti-DNA Abs synthesis was unaffected by this treatment, suggesting a selective immunosuppressive role in the anti-α4-integrin Ab.

The role of lymphokines in leukocyte recruitment to inflammatory sites has been well documented. The increased expression of counter-receptors for leukocyte adhesion proteins, such as intercellular cell adhesion molecule (ICAM-1), endothelial cell adhesion molecule (ELAM-1) and vascular cell adhesion molecule (VCAM-1) on EC, has been identified in sites of inflammation, including target organs for autoimmunity.[6,7]

Cytokines (especially TNF-α, IL-1 and IFN-γ) activate endothelial cells to synthesize and to increase the expression of adhesion molecules and conversely, engagement of these molecules on the surface of such cells can induce and/or mediate cytokine expression.[7,8] Recently, treatment of autoimmune diseases with cytokine agonists and their antagonists has been attempted in order to establish their clinical usefulness in the prevention of these disorders. Inflammatory mediators, such as TNF-α and IL-1β, have been

© 1995 Rapid Science Publishers extensively investigated in autoimmune diseases and their potential pro-inflammatory effects demonstrated by increasing the severity of the glomerular injury in both rats and rabbits in the anti-GBM antibody mediated model of nephritis.[9,10] In addition, in the renal cortices of lupus nephritic mice an enhancement of mRNA for TNF-α and IL-1β has been observed.[11] Also, an augmented production of TNF has been demonstrated in the nephritic glomeruli in anti-GBM glomerulonephritis (GN).[12] Moreover, it has been shown that the administration of anti-TNF antiserum abrogated the development of nephrotoxic nephritis in the rat.[13–15] Taken together, these data suggest that TNF-α and IL-1β can be regarded as potential mediators of glomerular injury, playing an important role in the induction and development of renal autoimmune diseases.

The presence of both TNF-α and VLA-α4-integrin has proved to be necessary for the development of proteinuria and accumulation of inflammatory cells in other rat models of nephritis,[15] unlike the requirements found in other models of renal diseases where complement and neutrophil-induced vascular injury are induced by deposition of immune complexes.[16]

Using the experimental model of autoimmune renal disease described in this paper, we have examined in depth the required presence of TNF-α for circulating leukocyte accumulation (in both glomerular and renal interstitium) as well as in the development of proteinuria.

### Materials and Methods

*Animals:* Brown Norway rats, weighing 180 to 200 g, were obtained from IFFA-CREDO (Paris, France) and from our own breeding colony, and maintained under standard conditions.

*Antibodies:* Rabbit anti-human TNF-α polyclonal antibody, which binds to human TNF-α and rat TNF-α,[17] was obtained from Genzyme Co. (Cambridge, MA). The mouse anti-human HP2/1 mAb recognizes the VLA-α4-integrin[18] and cross-reacts with the rat VLA-α4.[19] This mAb blocks the interaction of both α4β1 and α4β7 integrins with its two known ligands, FN and VCAM-1.[20,21] The following antibodies were purchased from Serotec (Oxford, UK): OX19, which recognizes the CD5 antigen;[22,23] W3/25, which reacts with a subset of peripheral T cells expressing the CD4 antigen;[24] OX8, which recognizes a stable determinant of the rat CD8 heterodimer;[25] OX6 mAb, which recognizes the rat MHC class II antigens (RT1-B);[26] and ED1 mAb, which reacts with rat monocytes and macrophages.[27]

*Experimental procedure:* Three groups of rats were injected s.c., three times a week, over a period of 2 weeks with 100 μg of $HgCl_2$ per 100 g/body wt.[28] Animals from Group I ($n = 20$) did not receive any additional treatment. Rats belonging to Group II ($n = 20$) received an i.p. injection (25 000 IU) of anti-TNF-α on days 0, 8 and 13 and those from Group III ($n = 20$) received an i.p. injection (0.5 mg) of HP2/1 mAb on days 0, 8 and 13. A fourth group (Group IV) ($n = 20$) served as a normal control in which rats were injected only with $H_2O$ adjusted to the same pH (3.8) as the $HgCl_2$ solution, following the same procedure described above for the mercury administration. The dosages and days were established on the basis of previous optimizing experiments and our appreciation of the kinetics of the disease.[5] All animals were sequentially bled on different days of the experiment by tail artery puncture. Four rats from each group were killed on days 4, 8, 11, 13 and 23 and kidneys were processed for study.

*Proteinuria:* Rats were maintained in metabolic cages for 24 h with free access to food and water. Urine samples were taken at regular intervals starting on day 0. Proteinuria was measured by using a Bio-Rad assay (Bio-Rad, Richmond, CA), according to the manufacturer's protocol. Urine samples were assayed in triplicate. The OD from each sample was measured in a Titertek Multiskan Plus (Flow, Irvine, UK) at 595 nm.

*Anti-GBM and anti-ssDNA Abs assay:* Rat GBM was isolated, essentially, as described by Bowman *et al.*[29] Briefly, glomeruli were obtained from normal BN rats by differential sieving and centrifugation of minced kidney cortices. The glomerular suspension was sonicated, washed and lyophilized. The GBM was digested with Type I collagenase (Sigma Chemical Co., St Louis, MO) at 0.7% w/w for 1 h at 37°C. Anti-GBM Abs were measured by ELISA as described previously.[23] Anti-ssDNA Abs were measured by an ELISA developed in our laboratory.[5] All the samples were assayed in quadruplicate.

Samples of a serum pool from untreated BN rats and from BN rats which were treated with $HgCl_2$ and bled on day 13 of the disease served as negative and positive controls, respectively. Results were expressed as the percentage of binding obtained with samples from positive control serum.

*Isolation and culture of glomeruli:* Harvested kidneys from the four experimental groups of rats were decapsulated and the renal medulla removed. The dissected cortex was then minced with a razor blade in Hanks' balanced salt solution (HBSS) and seived through decreasing pore size (250, 150 and 75 μm) as described previously.[30] Glomeruli were finally collected on the top of a 75 μm sieve, with the suspension containing more than 95% of glomeruli free of tubular fragments. Preparations were suspended at a final concentration of 5 000 glomeruli/ml in RPMI 1640 (Whittaker Bioproducts, Walkerville, MD) containing 10% (v/v) FCS (Flow), ampicillin (100 μg/ml) and streptomycin (100 μg/ml), and then incubated in 24-well plates (Nunc, Roskilde, Denmark) (5 000 glomeruli/well) at 37°C in a 5% $CO_2$ atmosphere. Supernatants were harvested at 24 h, centrifuged at 100 **g** and stored at −20°C until assayed for the release of TNF.

All samples were screened for the presence of endotoxin by using the *Limulus amoebocyte* lysate (L.A.L.) assay (Whittaker). Briefly, dilutions were performed from standard endotoxin (0.5 to 0.03 E.U./ml, 1 E.U. = 0.1 ng/ml of endotoxin) as positive control as well as for our samples (using serial dilutions, 1:2 to 1:64). Non-pyrogenic water was used as negative control. All samples were assayed in quadruplicate. One hundred μl of L.A.L. (sensibility, 0.1 E.U./ml) was added to each sample and then were incubated at 37°C for 1 h. The endotoxin concentration was obtained as the product of the lysate sensibility per the 'limit point dilution' (the last dilution showing reaction with the L.A.L.). The limit of detection in the assay was 10 pg/ml, with all the samples screened showing less than 10 pg/ml.

*TNF-α assay:* The TNF-α activity was determined by standard MTT method on L929 cells (ATCC, Rockville, MD)[31] in the presence of actinomycin D (1 μg/ml). Briefly, $3 \times 10^4$ cells/well were resuspended in DMEM (Whittaker) containing 10% (v/v) FCS, ampicillin (100 μg/ml), streptomycin (100 μg/ml), and glutamine (10 mM) and further incubated overnight on flat-bottomed, 96-well plates (Nunc) at 37°C. Dilutions of supernatants from cultured glomeruli were added in triplicate. Also, half-log dilution of hrTNF (specific activity, $2 \times 10^7$ to $2 \times 10^9$ units/ml) (Genzyme) ranging from 200 μg/ml to 1 pg/ml was added to some wells to produce a standard curve. A 25 μl aliquot of MTT stock solution (5 mg/ml) was added to each of the wells and incubated for 4 h at 37°C. The supernatants were removed by careful aspiration and 200 μl of a 1:1 DMSO and ethanol mixture to solubilize the crystals was then added to each well. The plates were shaken gently and OD (to 595 nm) was determined using a Titertek Multiskan Plus.

For neutralization studies, anti-TNF-α Ab or control sera were added simultaneously to wells containing 400 pg/ml of hrTNF-α (producing 50% lysis of $3 \times 10^4$ L929 cells, in our experiments) or test samples, with a final concentration of 50% (v/v). This Ab neutralizes 2 000 U of rTNF-α.[31] No TNF-α activity was found on L929 cells in those wells incubated with anti-TNF-α Abs.

Concentrations of TNF-α were extrapolated from a standard curve with known hrTNF-α dilutions. Results are expressed as pg/ml of TNF.

*Kidney tissue processing:* On days 13 and 23, rat kidneys ($n = 4$) from the groups I, II and IV were processed for histological and immunohistochemistry studies. For light microscopy, 3 μm paraffin-embedded kidney sections were stained with periodic acid–Schiffs (PAS). For immunohistochemistry studies, pieces of renal tissue were snap-frozen in isopentane precooled in liquid nitrogen, and stored at −70°C until used. Direct immunofluorescence studies were performed on ether/ethanol-fixed serial cryostat sections, by using FITC-conjugated rabbit anti-rat IgG (Serotec), as described previously.[32] In addition, the glomerular cell infiltrates were characterized in the kidneys of rats ($n = 4$) injected with mercury and in two control animals on days 4, 8, 13 and 23. These tissue kidneys were stained with an indirect immunophosphatase method (APAAP)[33] using a panel of mAbs. The specificity of these mAbs was assessed by using normal serum, normal mouse IgG, and hybridoma-induced ascitic fluids containing unrelated Abs. Positive controls of the reagents were sections of normal rat spleen. These studies were performed by using a conventional light microscopy objective (× 63), as described previously.[34]

*Statistical analysis:* The results are given as mean ± S.D. Values obtained from the levels of proteinuria and ELISA results were analysed using the Student's *t*-test. For TNF assay, statistical analysis was performed by using a Wilcoxon rank-sum method for non-parametric significance testing.

### Results

*Effect of anti-TNF-α treatment on the proteinuria:* As shown in Fig. 1, rats belonging to the Group I developed proteinuria in two different phases. A first short phase, which occurred immediately after the first injection of $HgCl_2$, due to the direct effect of mercury on tubular renal cells. This first phase was followed by a second phase starting on day 11 and declining after day 16 of the disease. On day 23, all the animals reached the background levels. When the rats

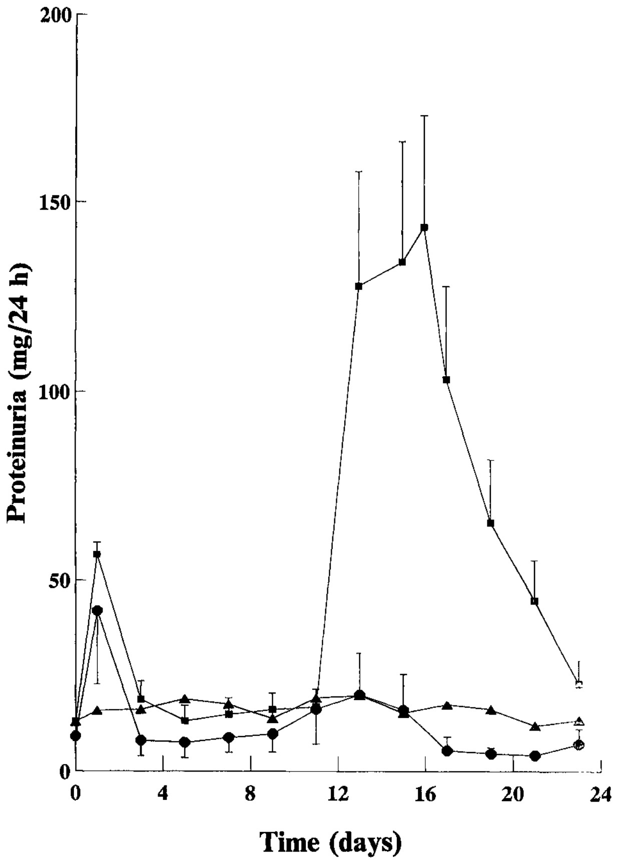

FIG. 1. Time course of urine protein excretion in normal control rats (Group IV) (▲) compared with $HgCl_2$-injected rats (Group I) (■) and anti-TNF-α-treated rats (Group II) (●). The results are expressed as mean ± S.D. Statistical analysis was performed by Student's *t*-test comparing Group I and II. *p*-values were <0.001.

were treated with anti-TNF-α Ab (Group II), a drastic reduction (about 90%, $p < 0.001$) in the urinary protein levels were found as compared with Group I ($20.21 \pm 7.99$ mg/24 h *vs.* $128.04 \pm 30.25$ mg/24 h). The same effect was observed when rats were treated with anti-α4 integrin Abs (HP2/1 mAb).[5] In both situations, the first phase of proteinuria was unaffected.

*Effect of anti-TNF-α treatment on anti-GBM and anti-ssDNA synthesis:* Increased anti-GBM Ab concentration in the serum from rats injected with mercury (Group I, Fig. 2A) was detected by ELISA from day 8, with the maximal concentration being observed on day 13 of the disease. After day 13, serum levels of anti-GBM Abs started to decline as also occurred with the proteinuria. The serum levels of anti-ssDNA Abs in this same Group I (Fig. 2B) showed a significant increase, first observed on day 4, but their kinetics of secretion were different from the anti-GBM Abs production.

In contrast to that found in BN rats treated with HP2/1 mAbs, which showed a significant ($p < 0.001$) reduction in the anti-GBM Abs serum levels,[5] the group of rats treated with anti-TNF-α Abs (Group II) presented circulating anti-GBM Abs levels similar to those found in Group I ($HgCl_2$-injected rats) (Fig. 2A). All the groups analysed, with the exception of the negative control group (Group IV), showed the same kinetics as found in the anti-ssDNA Abs synthesis

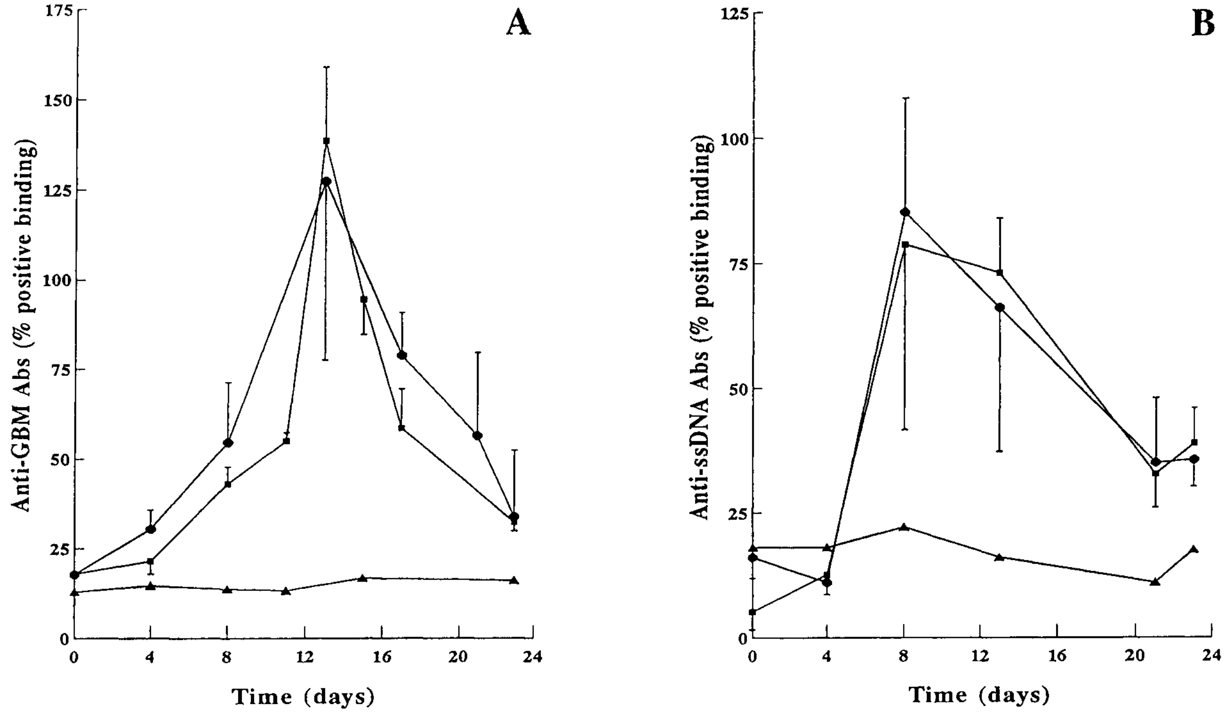

FIG. 2. Levels of circulating autoantibodies. Titre of serum anti-GBM Abs (A) and anti-ssDNA Abs (B) during the experiment in normal rats (Group IV) (▲) compared with $HgCl_2$-injected rats (Group I) (■) and anti-TNF-α-treated rats (Group II) (●). The values are given as mean ± S.D.

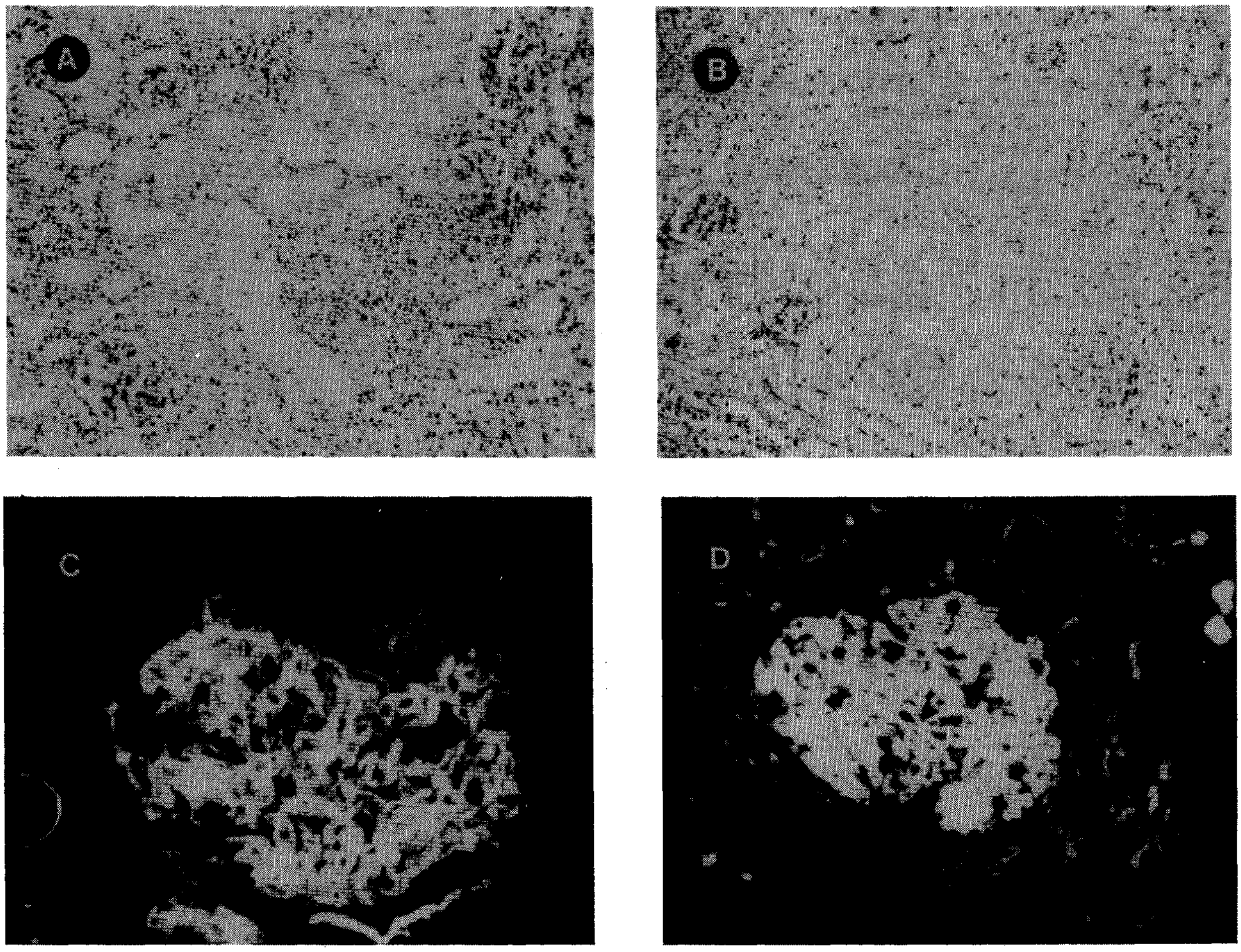

FIG. 3. Interstitial inflammatory cells accumulation in $HgCl_2$-treated rats (Group I) (A) showing severe perivascular renal cell infiltrates. Group of rats treated with anti-TNF-α Abs (Group II) (B) shows the absence of inflammatory cells in the renal interstitium (PAS, ×20). Both groups (I and II) (C, -IF ×500; and D, -IF ×425; respectively) show positive linear GBM deposits of rat IgG.

(Fig. 2B). None of the treatments affected the ssDNA Abs production.

*Histopathology and immunofluorescence studies on kidneys:* Renal tissues from rats treated with $HgCl_2$ (Group I) and $HgCl_2$ plus anti-TNF-α Abs (Group II) ($n = 4$ rats/group), were examined by light microscopy on days 13 and 23 of the disease (Fig. 3A and B). As reported previously,[3,5] kidney tissues from BN rats injected with mercury presented a severe interstitial mononuclear cell infiltrate. The inflammatory cells were preferentially located in the perivascular regions of the renal interstitium. However, in the same group of rats treated with anti-TNF-α Abs, cell infiltrates were not observed in the renal interstitium (Fig. 3B). Kidneys from $H_2O$-injected rats showed a normal renal histology.

Phenotypic analysis of glomerular cell infiltrates are given in Table 1. We found an impor-

**Table 1.** Glomerular leukocytes during $HgCl_2$-induced nephritis

| | Number of positive cells/glomerulus† | | | | |
|---|---|---|---|---|---|
| mAb | 0 | 4 | 8 | 13 | 23 |
| OX6 | 0.40 ± 0.07‡ | 0.65 ± 0.15 | 1.56 ± 0.60* | 1.53 ± 0.50* | 0.33 ± 0.15 |
| ED1 | 0.30 ± 0.06 | 0.67 ± 0.03* | 1.30 ± 0.28* | 0.50 ± 0.21 | 0.27 ± 0.10 |
| OX8 | 0.22 ± 0.07 | 0.20 ± 0.05 | 0.17 ± 0.06 | 0.70 ± 0.10* | 0.20 ± 0.03 |

†Six rats, and two sections per rat were examined for each mAb. Twenty glomeruli were counted per section.
‡Result are expressed as mean ± S.D.
*$p < 0.01$.

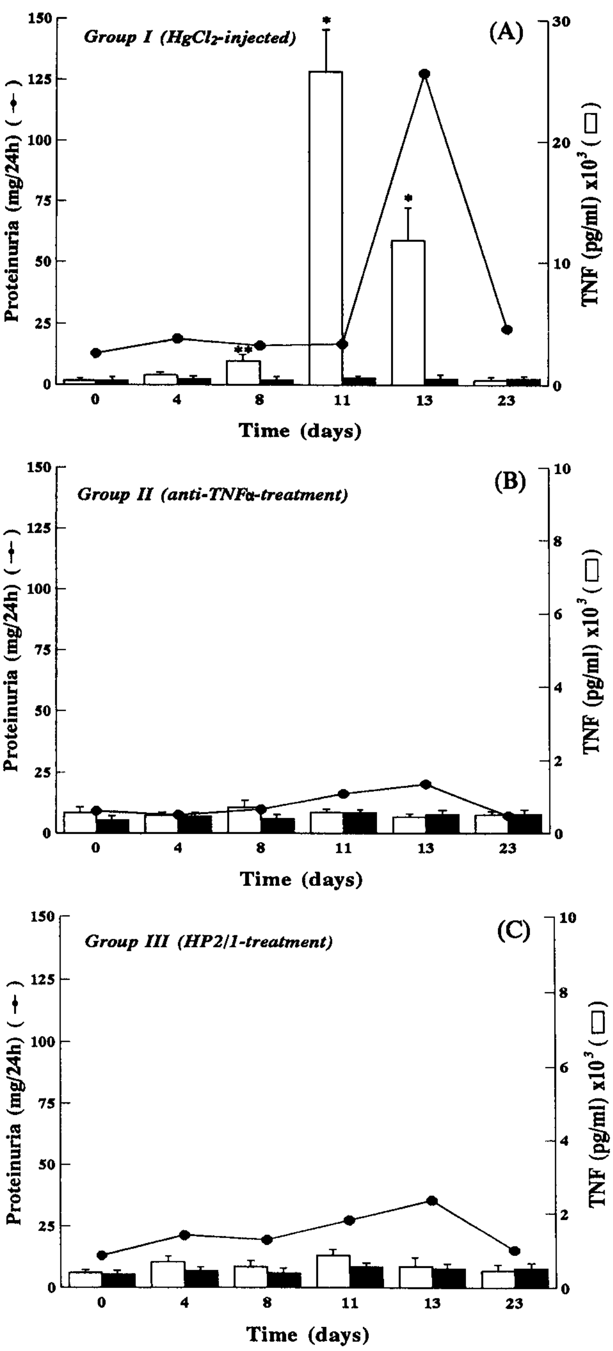

FIG. 4. TNF-α concentration from glomerular cultured supernatants. A. Glomerular TNF-α production (open bar) from $HgCl_2$-injected rats (Group I) and relationship between the development of proteinuria (●). B and C show the results obtained from glomerular cultured supernatants belonging to anti-TNF-α treated rats (Group II) and anti-α4 integrin (HP2/1)-treatment rats (Group III), respectively. Filled bar represents the TNF-α concentration from glomerular cultured supernatants from control rats (Group IV). Results shown represent the mean ± S.D. $^{*}p < 0.01$, and $^{**}p < 0.05$.

tant increase in the number of monocyte/macrophage ($ED1^+$ cells) on day 4 (0.67 cells/glomerulus) as compared with day 0 (0.30 cells/glomerulus), with this increase being maximal on day 8 (1.30 monocytes/glomerulus). Nevertheless, on day 13 of the disease, the number of monocytes decreased, and $CD8^+$ lymphocytes ($OX8^+$ cells) had significantly increased in comparison with the number on day 0 (0.22 cells/glomerulus and 0.70 cells/glomerulus, respectively). Both monocytes ($ED1^+$) or $CD8^+$ lymphocytes ($OX8^+$), in the glomerular cell infiltrates, also bear the $Ia^+$ antigen cell marker (Table 1).

On the other hand, when rat kidney tissues were examined by direct immunofluorescence studies, rat IgG showed a positive linear pattern deposition along the GBM in both $HgCl_2$-injected and anti-TNF-α-treated rats (but not control rats), at day 13 of the disease (Fig. 3C and D).

*Kinetics of TNF-α secretion by culturing glomeruli:* TNF-α concentration from glomerular cultured supernatants was measured after 24 h incubation by using a bioassay. As shown in Fig. 4A, the TNF-α levels in supernatants from cultured $HgCl_2$-injected rat (Group I) glomeruli started to increase before day 8 of the disease, reached a peak on day 11 ($p < 0.01$), and then it declined by day 12. Figure 4A illustrates both the kinetics of TNF-α production and the urinary protein excretion. Interestingly, TNF-α production preceded the development of proteinuria by 2–3 days. When TNF-α production was analysed in glomeruli isolated from rats belonging to both Groups II (anti-TNF-α treated) and III (anti-α4 treated), no significant levels of this cytokine was detected (Fig. 4B and C), in accordance with the low urinary protein levels.

### Discussion

The autoimmune disease induced in the BN rats by the injection of $HgCl_2$ is characterized by the synthesis of autoantibodies (mainly, anti-GBM Abs) due to a polyclonal B cell activation. The renal lesions consist of rat Ig deposition on the glomerular basement membrane with an influx of mononuclear cells and the development of proteinuria.[1–4] The administration of anti-TNF-α antiserum to $HgCl_2$-injected rats significantly reduced the urinary protein levels but the synthesis of anti-GBM Abs as well as glomerular deposits of Ig remained unaffected. In addition, no infiltrating interstitial cells were found in this group of rats. These findings suggest that TNF-α plays an important role in the development of this renal injury, as has been demonstrated previously in other different experimental models of nephritis.[14,15]

The elevated serum production of autoantibodies observed after anti-TNF-α treatment strongly suggests that this antiserum acts mainly at a local level. More evidence supporting this local anti-TNF-α Abs effect was obtained when we studied the production of TNF-α from isolated nephritic glomeruli. The glomeruli from $HgCl_2$-treated rats secreted TNF-α between days 4–8 to the day 11 of the disease. Also, the studies carried out on the glomerular cell infiltrates in $HgCl_2$-injected rats demonstrated an influx of monocytes on day 4 of the disease with the highest number of infiltrating glomerular cells having been observed on day 8. On the other hand, an increase in the number of $CD8^+$ lymphocytes was seen in the nephritic glomeruli on day 13. Moreover, the treatment with the anti-α4 integrin mAb to $HgCl_2$-injected rats completely abrogated the secretion of TNF-α from rat glomeruli. The α4 integrin, an adhesion molecule expressed by almost all leukocytes, interacts with VCAM-1 (an endothelial pro-inflammatory inducible cell molecule), as well as with the alternative spliced form (CS-1) of FN, playing a central role in mediating leukocyte adhesion, extravasation, and migration to sites of inflammation.[35,36] As has been previously demonstrated,[5] the administration of anti-α4 integrin mAb to $HgCl_2$-injected rats blocked the influx of circulating leukocytes into the renal interstitium and the accumulation of monocytes in the renal glomeruli. These data might suggest that infiltrating glomerular monocytes are the major source of glomerular TNF. The results presented here are in agreement with those previously reported by Tipping *et al.*[12] demonstrating the association between the glomerular monocyte infiltration and glomerular injury in anti-GBM nephritis. Nevertheless, the initial source of TNF in the glomerulus is still not entirely understood. It is well documented that resident glomerular cells can be responsible for TNF secretion. The stimulation of mesangial cells with LPS induces the synthesis of TNF-α,[37] and the *in vivo* administration of LPS also induces glomerular TNF-α mRNA expression in the absence of leukocyte cells infiltration.[38] Also, it has been shown that stimulated resident glomerular macrophages have the faculty to release cytokines such as IL-8, GM-CSF and TNF-α, among others.[39]

It is well known that inorganic mercury remains a major environmental toxin that alters cell calcium homeostasis[40] and mitochondrial functions.[41] In concentrations commonly used in experimental models the $Hg^{2+}$ acts as an ionophore as well as $Cu^+$.[42,43] In addition, the administration of $HgCl_2$ causes the activation of circulating lymphocytes, resulting in a polyclonal B-cell activation[2] and also producing a direct toxic effect on tubular renal cells. It is possible that mercury also has the potential to induce the activation of resident glomerular cells, thus initiating the secretion of cytokines as TNF-α. This local secretion of cytokines, in addition to stimulating chemotaxis, can induce the recruitment of circulating monocytes throughout the VLA-4/VCAM-1 cell adhesion pathway. The mechanism proposed could be supported based on the low levels of TNF-α in the supernatants of cultured glomeruli and on the absence of renal tissue cell infiltrates after mercury-injected rats were treated with the anti-α4 integrin.

### References

1. Sapin D, Druet E, Druet P. Induction of anti-glomerular basement membrane antibodies in the Brown Norway rat by mercuric chloride. *Clin Exp Immunol* 1977; **28:** 173–179.
2. Hirsch F, Couderc J, Sapin C, Fournie G, Druet P. Polyclonal effect of $HgCl_2$ in the rat, its possible role in an experimental autoimmune disease. *Eur J Immunol* 1982; **12:** 620–625.
3. Aten J, Bosman CB, Rozing GJ, Stijnen T, Hoedemaeker PJ, Weening GJ. Mercuric chloride-induced autoimmunity in the Brown Norway rat. Cellular kinetics and MHC antigen expression. *Am J Pathol* 1988; **133:** 127–138.
4. Pusey CD, Bowman C, Morgan A, Weetman AP, Hartley B, Lockwood CM. Kinetics and pathogenicity of autoantibodies induced by mercuric chloride in Brown Norway rats. *Clin Exp Immunol* 1990; **81:** 76–82.
5. Molina A, Sánchez-Madrid F, Bricio T, *et al.* Prevention of mercuric chloride, induced nephritis in the Brown Norway rat by treatment with antibodies against the α4 integrin. *J Immunol* 1994; **153:** 2313–2320.
6. Hartung HP. Immune-mediated demyelination. *Ann Neurol* 1993; **33:** 563–569.
7. Pober JS, Cotran RS. What can be learned from the expression of the endothelial adhesion molecules in tissue? *Lab Invest* 1991; **64:** 301–305.
8. Schattner A. Lymphokines in autoimmunity. A critical review. *Clin Immunol Immunopathol* 1994; **70:** 177–189.
9. Tomosugi NI, Cashman SJ, Hay H, *et al.* Modulation of antibody-mediated glomerular injury *in vivo* by bacterial lipopolysaccharide, TNF, and IL-1. *J Immunol* 1989; **142:** 3083–3090.
10. Bertani T, Abbate M, Zoja C, *et al.* Tumor necrosis factor induces glomerular damage in the rabbit. *Am J Pathol* 1989; **134:** 419–430.
11. Brennan DC, Yui MA, Wuthrich RP, Kelley VE. Tumor necrosis factor and IL-1 in New Zealand black/white mice, enhanced gene expression and acceleration of renal injury. *J Immunol* 1989; **143:** 3470–3475.
12. Tipping PG, Leong TW, Holdsworth SR. Tumor necrosis factor production by glomerular macrophages in anti-glomerular basement membrane glomerulonephritis in rabbits. *Lab Invest* 1991; **65:** 272–279.
13. Hruby ZW, Shirota K, Jothy S, Lowry RP. Antiserum against tumor necrosis factor α and protease inhibitor reduce immune glomerular injury. *Kidney Int* 1991; **40:** 43–51.
14. Karkar MA, Koshino Y, Cashman SJ, *et al.* Passive immunization against TNF-α and IL-1β protects from LPS enhancing glomerular injury in nephrotoxic nephritis in rats. *Clin Exp Immunol* 1992; **90:** 312–318.
15. Mulligan MS, Johnson KJ, Todd RF, *et al.* Requirements for leukocyte adhesion molecules in nephrotoxic nephritis. *J Clin Invest* 1993; **91:** 577–587.
16. Mulligan MS, Varani J, Dame MK, *et al.* Role of ELAM-1 in neutrophil-mediated lung injury in rats. *J Clin Invest* 1991; **88:** 1396–1406.
17. Chin YH, Cai JP, Johnson K. Lymphocyte adhesion to cultured Peyer's Patch high endothelial venule cells is mediated by organ-specific homing receptors and can be regulated by cytokines. *J Immunol* 1990; **145:** 3669–3677.
18. Sánchez-Madrid F, de Landázuri MO, Morago G, Cebrián M, Acevedo A, Bernabeu C. VLA-3: a novel polypeptide association within the VLA molecular complex: cell distribution and biochemical characterization. *Eur J Immunol* 1986; **16:** 1343–1349.
19. Yednock TA, Cannon C, Fritz LC, Sánchez-Madrid F, Steinman L, Karin N. Prevention of experimental autoimmune encephalomyelitis by antibodies against α4β1 integrin. *Nature* 1992; **356:** 63–66.
20. Pulido R, Elices MJ, Campanero MR, *et al.* Functional evidence for three distinct and independently inhibitable adhesion activities mediated by the human integrin VLA-4. *J Biol Chem* 1991; **266:** 10241–10245.
21. Postigo AA, Sánchez-Mateos P, Lazarovits AI, Sánchez-Madrid F, de Landázuri MO. α4β7 integrin mediates B cell binding to fibronectin and VCAM-1. Expression and function of α4 integrins on human B lymphocytes. *J Immunol* 1993; **151:** 2471–2483.
22. Eddy AA, Gary GS, Michael AF. Identification of lymphohaematopoietic cells in kidneys of normal rats. *Am J Pathol* 1986; **129:** 335–342.

23. Barclay AN. The localization of populations of lymphocytes defined by monoclonal antibodies in rat lymphoid tissues. *Immunology* 1981; **42:** 593–600.
24. Williams AF, Galfree G, Milstein C. Analysis of cell surfaces by xenogeneic myeloma-hybrid antibodies: differentiation antigens of rat lymphocyte. *Cell* 1977; **12:** 663–673.
25. Brideau RJ, Carter PB, McMaster WR, Mason DW, Williams AF. Two subsets of rat T lymphocyte defined with monoclonal antibodies. *Eur J Immunol* 1980; **10:** 609–615.
26. McMaster WR, Williams AF. Identification of Ia glycoproteins in rat thymus and purification from rat spleen. *Eur J Immunol* 1979; **9:** 426–433.
27. Dijkstra CD, Döpp EA, Joling P, Kraal G. The heterogeneity of mononuclear phagocytes: distinct macrophages subpopulations in the rat recognized by monoclonal antibodies ED1, ED2 and ED3. *Immunology* 1985; **54:** 589–599.
28. Bowman C, Mason DW, Pusey CD, Lockwood CM. Autoregulation of autoantibody synthesis in $HgCl_2$ nephritis in the BN rat. I. A role for T suppressor cells. *Eur J Immunol* 1984; **14:** 464–470.
29. Bowman C, Peters DK, Lockwood CM. Anti-glomerular basement membrane autoantibodies in the BN rat: detection by a solid-phase radioimmunoassay. *J Immunol Methods* 1983; **61:** 325–333.
30. Burlington H, Cronquite EP. Characteristics of cell cultures derived from renal glomeruli. *Proc Soc Exp Biol Med* 1973; **142:** 143–149.
31. Ju ST, Ruddle NH, Strack P, Dorf ME, DeKruyff RH. Expression of two distinct cytolytic mechanisms among murine CD4 subsets. *J Immunol* 1990; **144:** 23–31.
32. Mampaso F, Egido J, Martínez-Montero JC, *et al.* Interstitial mononuclear cell infiltrates in experimental nephrosis: effect of PAF antagonist. *Nephrol Dial Transpl* 1989; **4:** 1037–1044.
33. Cordell JL, Falini B, Erber WN, *et al.* Immunoenzymatic labelling of monoclonal antibodies using immune complexes of alkaline phosphatase and monoclonal anti-alkaline phosphatase (APAAP complex). *J Histochem Cytochem* 1984; **32:** 229–239.
34. Mampaso F, Wilson C. Characterization of inflammatory cells in autoimmune tubulointerstitial nephritis in rats. *Kidney Int* 1983; **23:** 448–457.
35. Hemler ME. VLA proteins in the integrin family: structures, functions and their role on leukocytes. *Ann Rev Immunol* 1990; **8:** 365–400.
36. Osborn L, Vasallo C, Benjamin C. Activated endothelium bind lymphocytes through a novel binding site in the alternatively spliced domain of vascular cell adhesion molecule-1. *J Exp Med* 1992; **176:** 99–107.
37. Affres H, Pérez J, Hagege J, *et al.* Desferrioxamine regulates tumor necrosis factor release in mesangial cells. *Kidney Int* 1991; **39:** 822–830.
38. Baud L, Oudinet JP, Bens M, *et al.* Production of tumor necrosis factor by rat mesangial cells in response to bacterial lipopolysaccharide. *Kidney Int* 1989; **35:** 1111–1118.
39. Brady HR. Leukocyte adhesion molecules and kidney diseases. *Kidney Int* 1994; **45:** 1285–1300.
40. Smith MW, Phelps PC, Trump BF. Cytosolic $Ca^{2+}$ deregulation and bleeding after $HgCl_2$ injury to cultured rabbit proximal tubule cells as determined by digital imaging microscopy. *Proc Natl Acad Sci USA* 1991; **88:** 4926–4930.
41. Lund BO, Miller DM, Woods JS. Studies on Hg(II)-induced $H_2O_2$ formation and oxidative stress *in vivo* and *in vitro* in rat kidney mitochondria. *Biochem Pharmacol* 1993; **45:** 2017–2024.
42. Tan XX, Tang C, Castoldi AF, L. Manzo, Costa LG. Effect of inorganic and organic mercury on intracellular calcium levels in rat T lymphocytes. *J Toxicol Environ Health* 1993; **38:** 159–170.
43. Karniski LP. $Hg^{2+}$ and $Cu^{2+}$ are ionophores, mediating $Cl^-/OH^-$ exchange in liposomes and rabbit renal brush border membranes. *J Biol Chem* 1992; **267:** 19218–19225.

ACKNOWLEDGEMENTS. This work was supported by Grants 92/0420 and 95/1420 from the Fondo de Investigaciones Sanitarias (FIS), Spain. We thank Ms Carmen Masa, Ms Mela López, and Ms Rosa Ocaña for technical assistance. We are grateful to Ms Virginia Lynne Desbrow for her help in the manuscript correction.

**Received 8 September 1995; accepted 16 October 1995**
